# New Books

**Published:** 2010-11

**America’s Environmental Report Card: Are We Making the Grade?**

Harvey Blatt

Cambridge, MA:MIT Press, 2010. 352 pp. ISBN: 978-0-262-51591-7, $19.95

**Assessment of Intraseasonal to Interannual Climate Prediction and Predictability**

Committee on Assessment of Intraseasonal to Interannual Climate Prediction and Predictability; National Research Council

Washington, DC:National Academies Press, 2010. 192 pp. ISBN: 978-0-309-15183-2, $43.25

**Climate Change and Food Security in South Asia**

K.R. Islam, A.H.M. Mustafizur Rahman, S.M.A. Faiz, M. Sivakumar, R. Lal, eds.

New York:Springer, 2011. 440 pp. ISBN: 978-90-481-9515-2, $209

**Climate Change Biology**

Lee Hannah

St. Louis, MO:Elsevier, 2010. 416 pp. ISBN: 978-0-12-374182-0, $59.95

**Coming Clean: Breaking America’s Addiction to Oil and Coal**

Michael Brune

San Francisco:Sierra Club Books, 2010. 288 pp. ISBN: 978-1-57805-190-8, $10.85

**Describing Socioeconomic Futures for Climate Change Research and Assessment**

Panel on Socio-Economic Scenarios for Climate Change Research and Assessment; National Research Council

Washington, DC:National Academies Press, 2010. 80 pp. ISBN: 978-0-309-16144-2, $21

**Energy Economics: CO2** **Emissions in China**

Y. Wei, L. Liu, G. Wu, L. Zou, eds.

New York:Springer, 2011. 360 pp. ISBN: 978-3-642-13846-1, $179

**Freshwater Ecology, 2nd ed.**

Walter K. Dodds, Matt R Whiles

St. Louis, MO:Elsevier, 2010. 829 pp. ISBN: 978-0-12-374724-2, $99.95

**Global Change: Mankind–Marine Environment Interactions**

H.-J. Ceccaldi, I. Dekeyser, M. Girault, G. Stora, eds.

New York:Springer, 2011. 450 pp. ISBN: 978-90-481-8629-7, $179

**Global Ecology**

Sven Erik Jorgensen, ed.

St. Louis, MO:Elsevier, 2010. 480 pp. ISBN: 978-0-444-53626-6, $79.95

**Health Hazards of Environmental Arsenic Poisoning: From Epidemic to Pandemic**

Chien-Jen Chen, Hung-Yi Chiou, eds.

Hackensack, NJ:World Scientific, 2010. 350 pp. ISBN: 978-981-4291-81-1, $124

**Impacts of Global Change on the Hydrological Cycle in West and Northwest Africa**

Peter Speth, Michael Christoph, Bernd Diekkrüger, eds.

New York:Springer, 2011. 675 pp. ISBN: 978-3-642-12956-8, $179

**In Extremis: Disruptive Events and Trends in Climate and Hydrology**

Jürgen Kropp, Hans-Joachim Schellnhuber, eds.

New York:Springer, 2011. 400 pp. ISBN: 978-3-642-14862-0, $169

**Ocean Acidification: A National Strategy to Meet the Challenges of a Changing Ocean**

Committee on the Development of an Integrated Science Strategy for Ocean Acidification Monitoring, Research, and Impacts Assessment; National Research Council

Washington, DC:National Academies Press, 2010. 175 pp. ISBN: 978-0-309-15359-1, $32

**Pathways to Urban Sustainability: Research and Development on Urban Systems**

Daniel Schaffer, Derek Vollmer, eds.

Hackensack, NJ:World Scientific, 2010. 124 pp. ISBN: 978-0-309-15895-4, $31.75

